# Differences in ginseng quality and soil characteristics in different growth modes of *Panax ginseng*

**DOI:** 10.3389/fpls.2026.1825327

**Published:** 2026-07-08

**Authors:** Keming Cao, Chanpeng Sun, Jia Tao, Linlin Zhang, Xiaoxi Pan, Yue Zhang, Cai Shao, Yiming Guan, Qiuxia Wang

**Affiliations:** Institute of Special Animal and Plant Science, Chinese Academy of Agricultural Science, Changchun, China

**Keywords:** cultivated ginseng, ginsenoside, soil, understory ginseng, wild ginseng

## Abstract

**Introduction:**

Ginseng has three main growth modes, and there are significant differences in quality among ginseng grown under these different modes; however, the mechanism by which soil nutrients and enzyme activity influence ginsenosides synthesis, leading to differences in quality among ginseng grown under different modes, remains unclear.

**Methods:**

We systematically measured soil physicochemical properties, soil enzyme activity, and total ginsenoside along with key monosaponin content in ginseng.

**Results:**

The results indicate that wild ginseng soil exhibited significantly higher total carbon, soil organic carbon, catalase, and sucrase activities compared to understory ginseng and cultivated ginseng, with generally higher available potassium levels; Soil from understory ginseng exhibited the highest acid phosphatase activity (60,546.74–71,212.00 U/g), with all indicators falling between wild ginseng and cultivated ginseng; cultivated ginseng soil had lower organic carbon content, and significantly lower urease activity than the other two growth patterns. Regarding ginsenoside content, wild ginseng exhibited the highest total ginsenoside content in both aboveground (7.74%) and belowground (5.28%) parts, with significantly superior levels of key monosides such as Rg1, F2, and Rh4 compared to both understory ginseng and cultivated ginseng. Correlation analysis revealed significant positive correlations between catalase and sucrase activities and total carbon and organic carbon content. Conversely, acid phosphatase activity showed a significant negative correlation with available potassium and phosphorus levels.

**Discussion:**

Taken together, the high organic matter content and synergistic high enzyme activity in wild ginseng soil provide ample substrates and energy for ginsenoside synthesis, constituting key factors for its superior quality. These findings provide theoretical support for optimizing ginseng cultivation practices and enhancing product quality.

## 1 Introduction

*Panax ginseng* Meyer is a perennial herbaceous plant of the family Araliaceae. Ginsenosides are the primary active constituents in ginseng, playing a crucial role in regulating the central nervous system, enhancing cardiac function, and influencing material metabolism. They are primarily applied in clinical treatments targeting the nervous system, cardiovascular system, and antitumor therapy (Geng et al., 2024; Fan and Wang, 2014). Currently, there are three primary growth modes for ginseng: wild ginseng, understory ginseng, and cultivated ginseng. Research has shown that wild ginseng and understory ginseng contain higher levels of ginsenosides than cultivated ginseng, primarily due to differences in their ecological environments (Yan et al., 2023). However, the differences in how soil properties affect the accumulation of active ingredients in ginseng grown under three cultivation methods remain poorly understood.

Wild ginseng refers to ginseng seeds sown in mountain forests by human and allowed to grow naturally. Understory ginseng involves seeds sown or seedlings transplanted into mountain areas with some level of human intervention. Cultivated ginseng is grown on man-made beds with human involvement throughout the entire growth process (Zhang, 2018). The quality of ginseng of these three modes varies significantly. As the medium for ginseng growth, soil directly or indirectly influences ginsenoside content through its nutrient levels and other properties. Researchers have found that soil N, P, K, and organic matter content are positively correlated with total ginsenoside content (Sun et al., 2012; Lin, 2016). Furthermore, although soil enzymes do not directly participate in the accumulation of ginseng’s active components, they serve as the driving force behind all biochemical reactions in the soil. By promoting the decomposition and transformation of organic matter and the release of available nutrients, soil enzymes also influence the accumulation of ginsenosides (Yang and Jiang, 2016). Researchers have also found that excessively high or low enzyme activity inhibits the accumulation of ginsenosides in ginseng (Yang and Jiang, 2016). Soil physicochemical properties and soil enzyme activity together form a dynamic biochemical network that synergistically regulates nutrient forms, transformation rates, and plant availability. Therefore, understanding the relationship between the accumulation of active ingredients in ginseng and soil properties is crucial. However, research on how soil nutrient content and soil enzyme activity influence ginseng under different cultivation modes remains scarce. Although previous studies have confirmed the correlation between individual or a few soil factors and the total ginsenoside content in ginseng, the pathways and mechanisms by which this series of key soil physicochemical indicators and diverse soil enzyme activities guide the synthesis and accumulation of ginsenosides of different growth modes remain unclear.

To investigate the effects of soil physicochemical properties on the differences in ginsenoside accumulation under different cultivation modes. Therefore, this study investigates the mechanism by which soil factors influence ginsenoside accumulation through the determination of soil indicators, total ginsenoside content, and the content of various ginsenosides, along with correlation analysis. The research findings will provide theoretical support for optimizing ginseng cultivation techniques and enhancing product quality.

## 2 Materials and methods

### 2.1 Sample collection and processing

**Figure 1:**
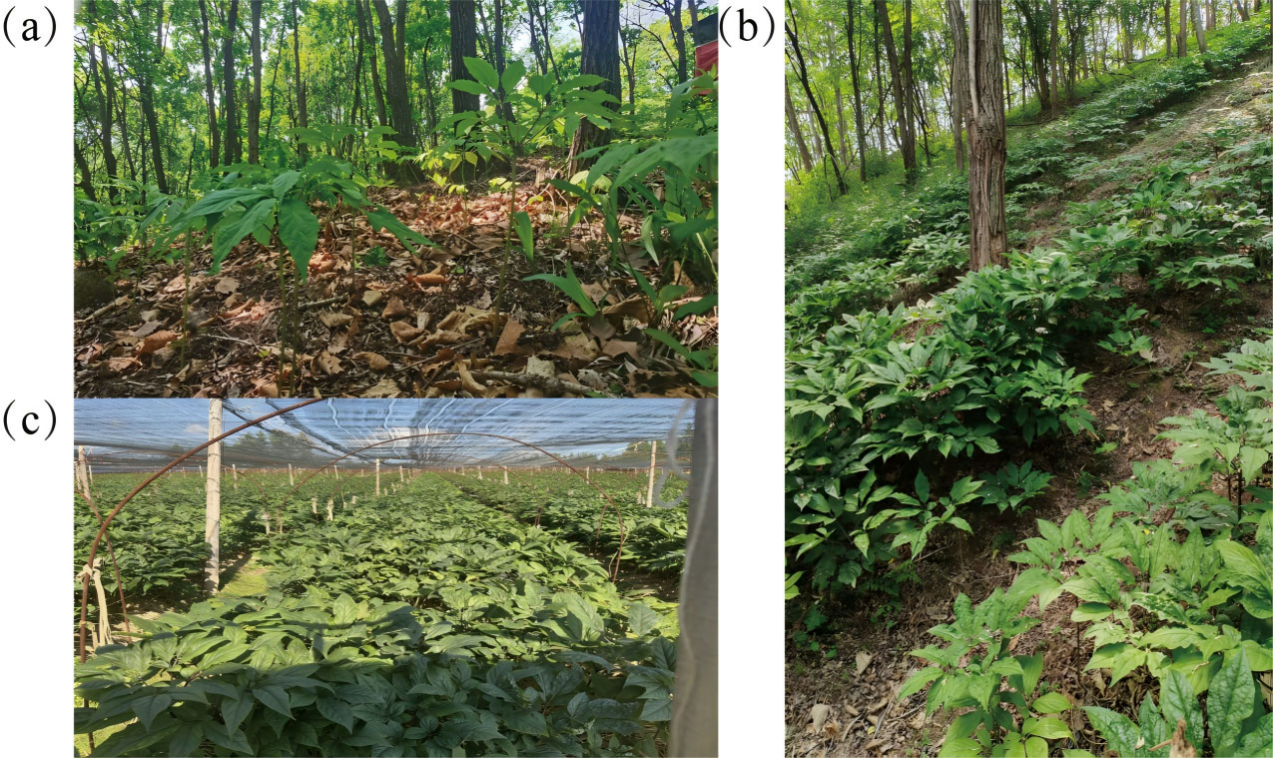
Wild ginseng habitat **(a)** Understory ginseng habitat **(b)** Cultivated ginseng habitat **(c)**.

Soil samples were collected from Yanji City, Wangqing County and Dunhua City in Yanbian Korean Autonomous Prefecture, Jilin Province, and Huanren Satisfaction Autonomous County in Benxi City, Liaoning Province, and the details of the samples are shown in **Tables 1**–**3**. Photographs of sampling points are shown in **Figure 1**. We use the five-point sampling method. Five biological replicates for each soil sample, with measurements repeated three times. The soil samples were collected 10–20 cm from the surface in sterile sampling bags after removing the layer of dead branches and leaves. Samples were stored in ice boxes and transported immediately to the laboratory. The samples were divided into two parts, one part was stored at 4 °C for the determination of soil enzyme activities and the remaining part was dried and passed through a 20 mesh sieve and a 100 mesh sieve for the determination of chemical properties of the soil samples.

**Table 1:** Information of wild ginseng soil samples.

No.	Latitude and longitude of sampling points	Orientation of the slope	Source of seeds	Years	Planting method	Companion tree species
YY1	43.167527°N 129.454698°E	North facing slightly easterly slope	Wild ginsengs harvested from Hunchun on the China-Russia border	35	seed sowing	linden,Acer mono,birch and toothed oak
YY2	43.169285°N 129.454375°E	east slope	Wild ginsengs harvested from Hunchun on the China-Russia border	11	seed sowing	linden,Acer mono,birch, toothed oak and Juglans mandshurica Maxim
YY3	43.171200°N 129.454767°E	east slope	Seeds from Huanren County	10	seed sowing	linden,Acer mono and birch
YY4	43.171345°N 129.454645°E	east slope	Seeds from Huanren County	10	seed sowing	linden,Acer mono and birch

**Table 2:** Information of understory ginseng soil samples.

No.	Latitude and longitude of sampling points	Orientation of the slope	Years	Planting Method	Companion tree species	Frequency of pesticide application	Frequency of fertilization
HRH1	41.348912°N 125.539487°E	south-west slope	20	seed sowing	larix gmellini	six times a yearr	once a year
HRJ2	41.332124°N 125.518367°E	north-west slope	20	seed sowing	oak and birch	six times a yearr	once a year
HRY3	41.331645°N 125.519172°E	south-west slope	24	seed sowing	Chinese pine and oak	three times a year	no fertilization
HRC4	41.332187°N 125.513502°E	north slope	19 + 8	Transplanting	oak and juglans mandshurica maxim	three times a year	no fertilization
HRL5	41.393041°N 125.464985E	north slope	12	seed sowin	Chinese scholar tree, pine, juglans mandshurica maxim and oak	three or four times a year	once a year

**Table 3:** Information of cultivated ginseng soil samples.

No.	Latitude of sampling points	Longitude of sampling points	Altitude of sampling points	Years
WN1	43.327394°N	129.749175°E	230.8 ± 8.43m	4
DN2	43.544250°N	127.942265°E	498.2 ± 7.85m	4
DN3	43.541267°N	127.871745°E	498.2 ± 7.85m	4
DN4	43.5418435°N	127.956465°E	498.2 ± 7.85m	4

Randomly select sampling points to collect ginseng samples. Each sample set contains five ginseng. Dig up the entire ginseng plant, place it into a labeled sampling bag, and transport it back to the laboratory. Upon arrival, wash the surface clean, separate the aboveground and underground parts, and allow them to air-dry naturally before testing. Wild ginseng samples are designated as YY1 and YY2. Understory ginseng samples are designated as HR1 and HR2. Cultivated ginseng samples are designated as N1 and N2. Samples with the number 1 in their ID number refer to the aboveground parts, while those with the number 2 refer to the belowground parts.

### 2.2 Determination of physical and chemical properties of soil

Soil total carbon (TC), total nitrogen (TN) content were determined using an elemental analyzer (EURO EA, China); NH_4_^+^-N and NO_3_^--^N, content were determined by AA3 continuous flow analyzer (EA3000, EuroVector, Australia); Soil available phosphorus (AP) content was determined using air-dried soil samples passed through a 20-mesh sieve. After extraction with sodium bicarbonate solution, molybdenum-antimony reagent was used as a color developer. Following a 30-minute incubation period, absorbance values were measured using a spectrophotometer (Bao, 2000). Soil available potassium (AK) content was determined using air-dried soil samples passed through a 20-mesh sieve. After extraction with ammonium acetate, the concentration was measured using a flame spectrophotometer (Bao, 2000).

### 2.3 Determination of soil enzyme activities

Soil catalase (S-CAT) was determined by potassium permanganate colorimetric titration (Wei et al., 2018); soil sucrase (S-SC) was determined by 3,5-dinitrosalicylic acid colorimetric assay (Xun et al., 2019); soil polyphenol oxidase (S-PPO) was determined by colorimetric method using o-phenyltriol (Zhang et al., 2022); soil acid phosphatase (S-ACP) was determined by disodium phenylphosphate colorimetric assay (You et al., 2015); soil urease (S-UE) was determined by indophenol blue colorimetric method (Xiao et al., 2016).

### 2.4 Metagenome sequencing, assembly, and functional annotation

Sampling 1 μg of genomic DNA, the samples were randomly fragmented into segments of approximately 350 bp using a Covaris ultrasonic disruptor to construct the library. The entire library preparation was completed through steps including end repair, addition of A-tails, ligation of sequencing adapters, purification, and PCR amplification. After library construction, the integrity of the library fragments and the size of the inserted fragments were assessed using AATI analysis. If the insert size meets expectations, the accurate concentration of the effective library was quantified using Q-PCR (effective library concentration > 3 nM) to ensure the library quality. After the library passed the quality check, different libraries were pooled according to their effective concentrations and target data output requirements, and then subjected to PE150 sequencing.

Fastp (https://github.com/OpenGene/fastp) was used for preprocessing raw data from the Illumina sequencing platform to obtain clean data for subsequent analysis. MEGAHIT software was used for assembly analysis of clean data, and scaftigs without N was obtained by breaking the resulted scaffolds from the N junction (Qin et al., 2010; Li et al., 2015). ORF prediction of sample scaftigs (≥500bp) was performed using MetaGeneMark (Karlsson et al., 2012; Mende et al., 2012; Li et al., 2014; Oh et al., 2014; Qin et al., 2014), filtering out information with lengths less than 100 nt (Qin et al., 2010; Zhu et al., 2010; Nielsen et al., 2014; Zeller et al., 2014), all with default parameters. The ORF prediction results were de-redundant with CD-HIT software (Li et al., 2006; Fu et al., 2012) to obtain a non-redundant initial gene catalogue. samples clean data were aligned to the initial gene catalogue using Bowtie2, and the number of reads on the gene alignment was calculated. Genes with ≤2 reads were filtered out to obtain the gene catalogue (unigenes) for subsequent analysis.

### 2.5 Determination of ginsenoside content

The ginsenoside content was determined by high-performance liquid chromatography, mainly including total ginsenoside, Rg1, Re, Rb1, CK, F2, R-Rh2, S-Rh2, R-Rh1, S-Rh1, Rk3, S-Rg3, R-Rg3, F4, Rk1, Rg5 and Rh4. Instrument Model: Shimadzu NexeRa XR-8045 Triple Quadrupole Liquid Chromatography-Mass Spectrometry System (HPLC-MS); Column Model: Agilent XDB-C18; Specifications: 3.5 μm, 2.1 × 100 mm; Temperature: 35 °C; Flow Rate: 0.2 mL/min; Detector: VWD; Wavelength: 203 nm; Mobile phase: acetonitrile and water.

### 2.6 Statistical analysis

Statistical analyses were performed using R 4.3.2 and Excel. Performing a significance analysis of differences using IBM SPSS Statistics 24. And images were drawn and jigsawed using Adobe Illustrator 2023. The bar chart, lollipop chart, matchstick chart and line chart were completed using R ggplot2, and the heatmap was completed using R pheatmap. Statistical analyses were performed using IBM SPSS Statistics 24. The data were first subjected to Levene test and the Kolmogorov-Smirnov test. One-way ANOVA was used for comparisons among multiple groups, assuming homogeneity of variances, followed by *post-hoc* pairwise comparisons using the Least Significant Difference (LSD) method. The *P* < 0.05 was considered statistically significant.

## 3 Results

### 3.1 Physical and chemical properties of soil

**Figure 2:**
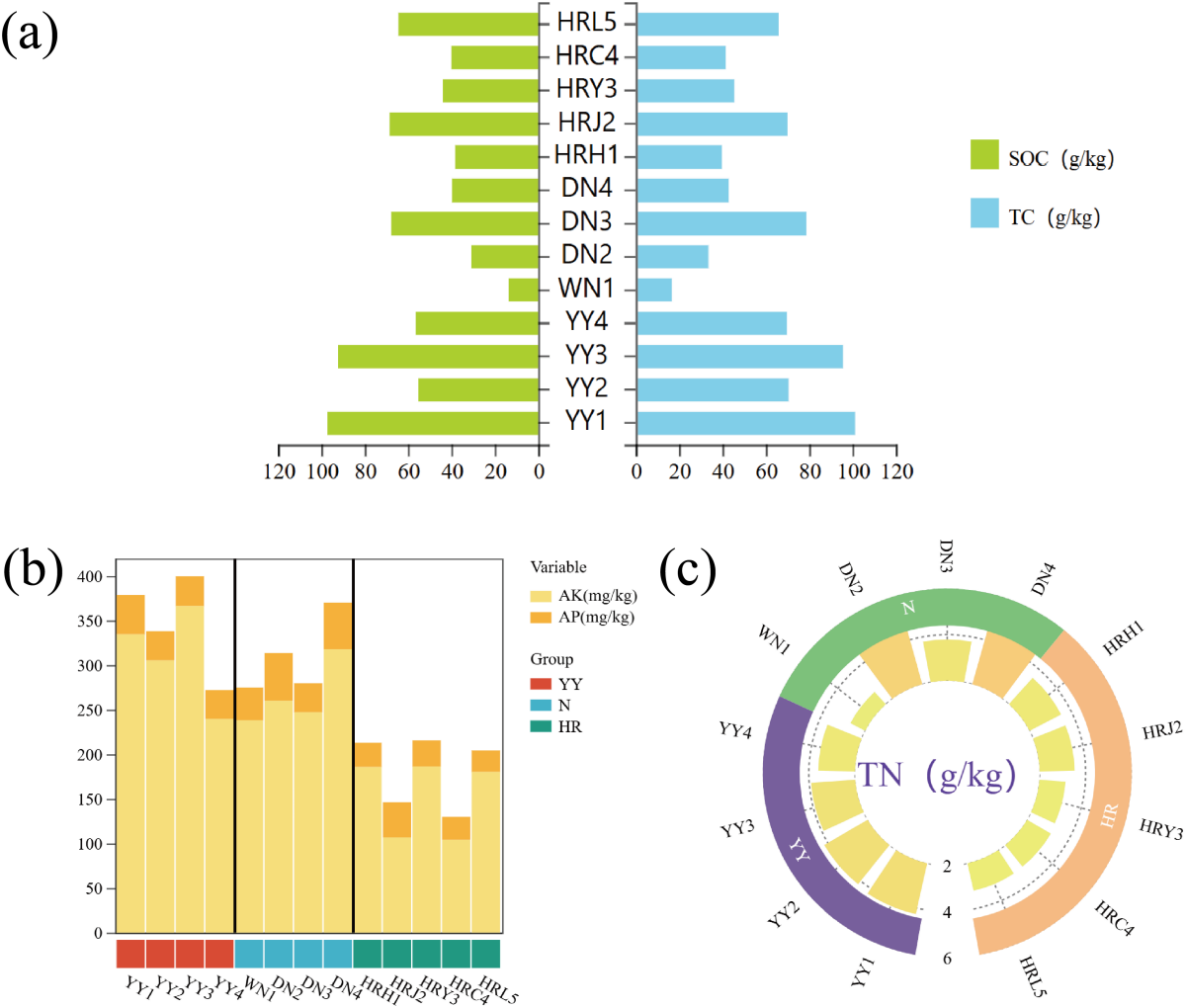
Soil SOC and TOC content of different growth modes of ginseng **(a)** Soil AK and AP content of different growth modes of ginseng **(b)** Soil TN content of different growth modes of ginseng **(c)**. Different lowercase letters in the same column represent significant differences.(*P* < 0.05).

The total carbon content in wild ginseng soil was higher than that in cultivated ginseng soil and understory ginseng soil. The range of total carbon content in wild ginseng soil, understory ginseng soil and cultivated ginseng soil were: 68.73 ~ 100.10 g/kg, 38.76 ~ 69.02 g/kg and 15.70 ~ 77.63 g/kg (**Figure 2a**). The Soil organic carbon (SOC) content of wild ginseng soil was extremely high, reaching 56.39 to 95.49 g/kg (**Figure 1a**). Differences in total nitrogen content were not significant among the three types of soil, the ranges of total nitrogen content in wild ginseng soil, understory ginseng soil and cultivated ginseng soil were 3.60 ~ 4.26 g/kg, 3.14 ~ 3.55 g/kg and 2.72 ~ 5.46 g/kg (**Figure 2c**). Except for a few samples, the available potassium (AK) content in wild ginseng soil (239.85 mg/kg ~ 366.43 mg/kg) was higher than that in cultivated ginseng soil (238.11 mg/kg ~ 317.92 mg/kg) (**Figure 2b**). The AP content in understory ginseng soil was the lowest, ranging from 104.25 mg/kg to 186.43 mg/kg. The available phosphorus (AP) content in wild ginseng soil and understory ginseng soil showed little difference. Except for HRJ2 (39.56 mg/kg), the AP content in understory ginseng soil (23.93 mg/kg–29.10 mg/kg) was lower than that in the other two modes (**Figure 2b**).

The numbers in parentheses represent the standard error.

### 3.2 Soil enzyme activity

**Figure 3:**
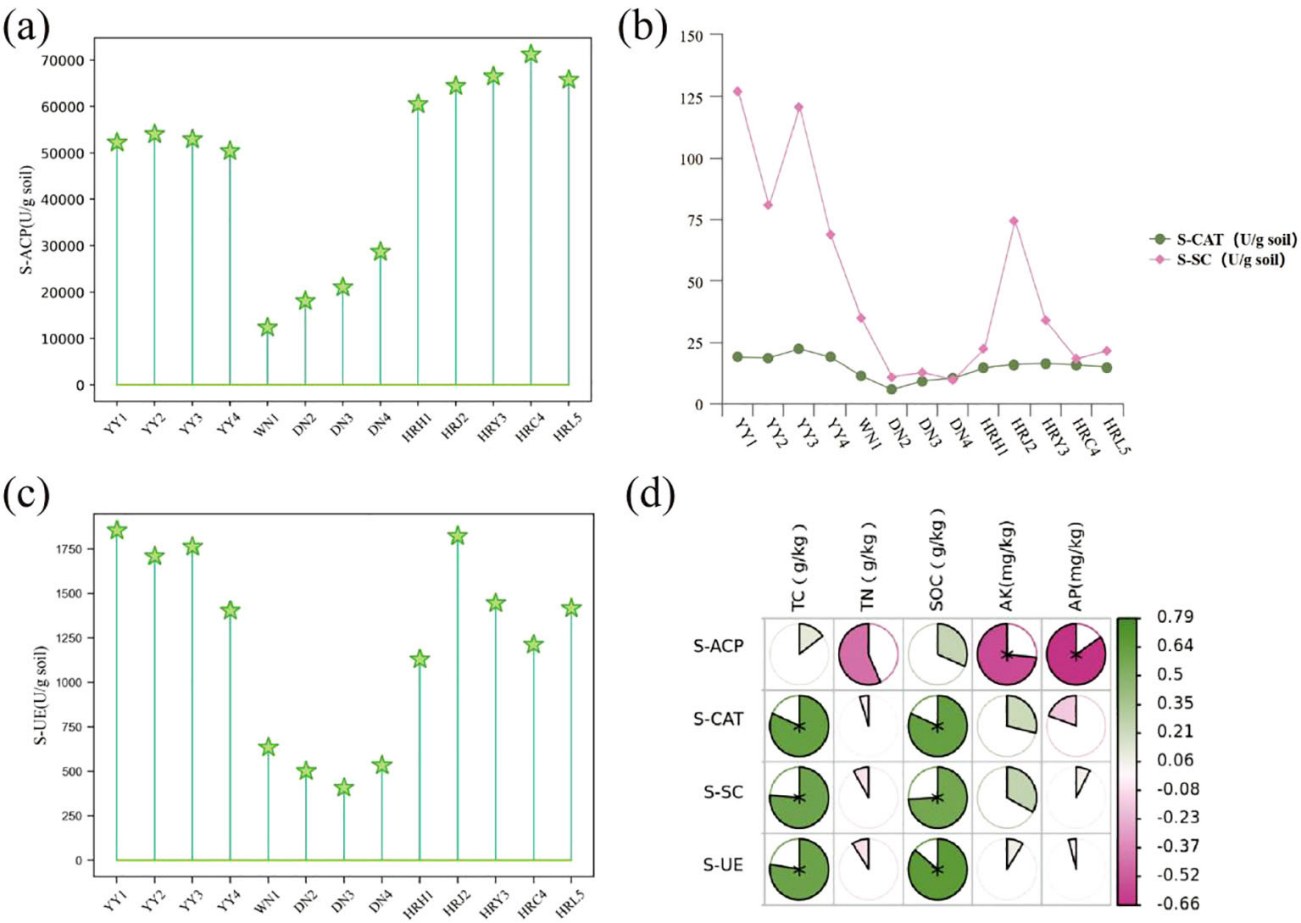
S-ACP in ginseng soil of different growth modes **(a)** S-CAT and S-SC in ginseng soil of different growth modes **(b)** S-UE in ginseng soil of different growth modes **(c)** Correlation analysis between soil enzyme activity and soil physicochemical properties **(d)**.

In soil of the three growth modes of ginseng, the order of S-ACP is: understory ginseng soil (60546.74U/g soil ~ 71212.00U/g soil) > wild ginseng soil (50,427.18 U/g soil ~ 54,077.43 U/g soil) > cultivated ginseng soil (12,352.46 U/g soil ~ 28,692.42 U/g soil), with extremely significant differences (*P* < 0.05) (**Figure 3a**). The S-CAT was ranked as follows: wild ginseng soil (18.36 U/g soil ~ 22.00 U/g soil) > understory ginseng (14.35 U/g soil ~ 16.05 U/g soil) > cultivated ginseng soil (5.40 U/g soil ~ 11.07 U/g soil), with extremely significant differences (*P* < 0.05) (**Figure 3b**). The S-SC in wild ginseng soil was significantly higher than in the other two modes (*P* < 0.05), ranging from 68.48 U/g soil to 126.86 U/g soil (**Figure 3b**). The S-UE in the cultivated ginseng soil was significantly lower than that in the other two modes (*P* < 0.05), ranging from 408.86 U/g soil to 634.45 U/g soil (**Figure 3c**).

### 3.4 Soil microorganisms and microorganism functional characteristics

**Figure 4:**
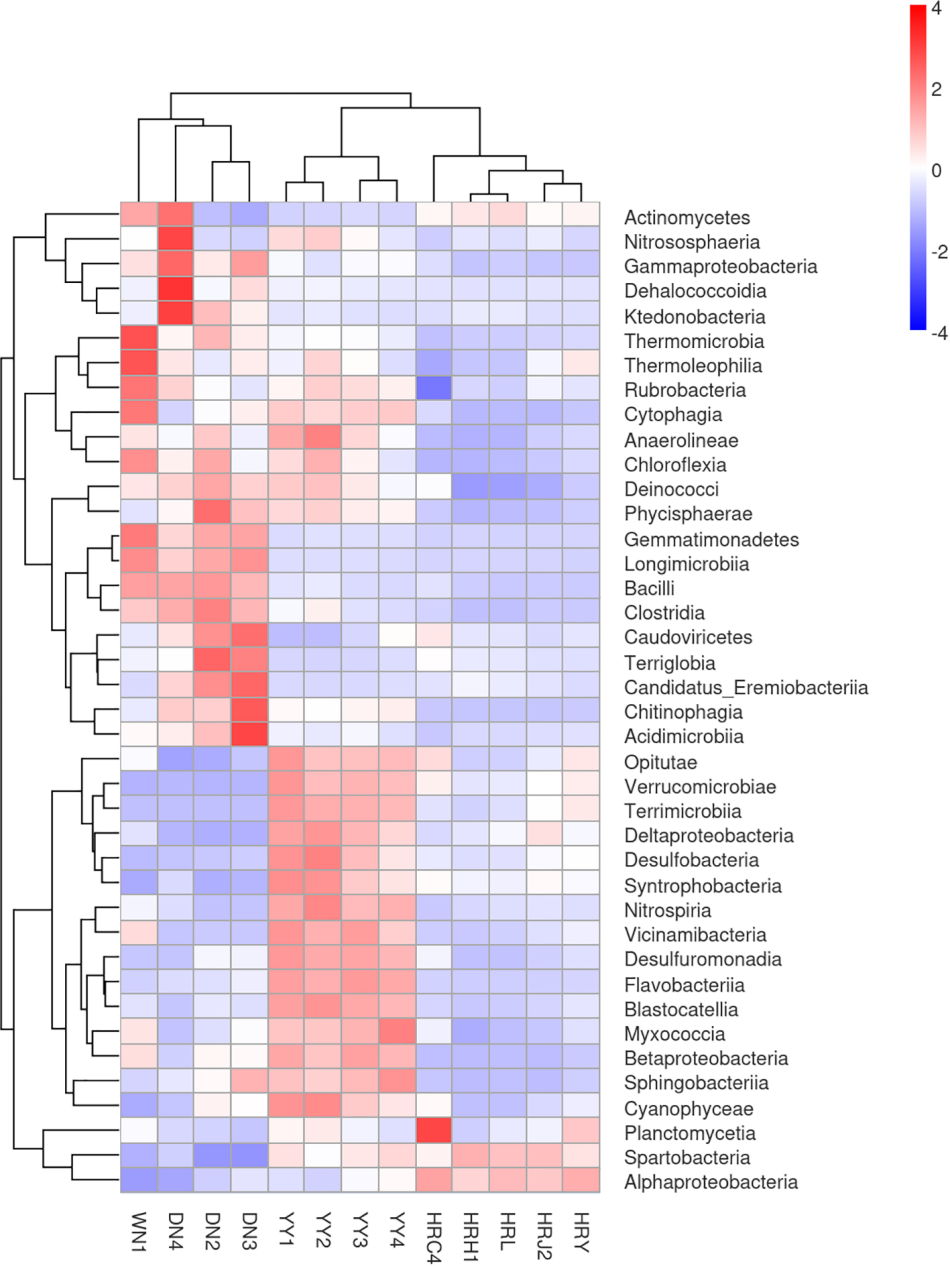
Clustering Heatmap of top 40 microorganisms at the class level in ginseng soil of different growth modes.

At the class level, samples from the same growth mode exhibited higher overall clustering and greater similarity in microbial community structure; samples from different growth modes showed distinct branching patterns, indicating that growth mode is the primary factor driving differences in microbial community structure. Compared with the other two growth modes, Opitutae, Verrucomicrobiae, Terrimicrobiia, Deltaproteobacteria, Desulfobacteria, Syntrophobacteria, Nitrospiria, Vicinamibacteria, Desulfuromonadia, Flavobacteriia, Blastocatelia, Myxococcia, Betaproteobacteria, Sphingobacteriia, and Cyanophyceae were significantly enriched in wild ginseng soil, while Spartobacteria and Alphaproteobacteria were significantly enriched in the understory ginseng soil, while the microorganisms significantly enriched in the cultivated ginseng soil included Gemmatimonadetes, Longimicrobiia, Bacilli, and Clostridia (**Figure 4**).

**Figure 5:**
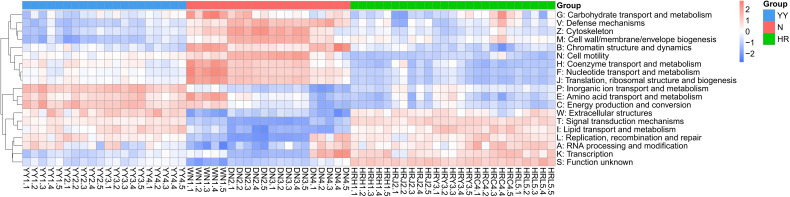
Clustering heatmap based on the eggNOG function category in ginseng soil of different growth modes.

Based on function annotation results from the eggNOG database, this study focused on analyzing function categories related to the biosynthesis of plant saponins. Among the function categories associated with saponin biosynthesis, function category C (Energy production and conversion) was highly abundant in wild ginseng soil, while function categories I (Lipid transport and metabolism) and T (Signal transduction mechanisms) were more abundant in wild ginseng soil and understory ginseng soil, function category K (Transcription) had a relatively higher abundance in understory ginseng soil, and function categories G (Carbohydrate transport and metabolism) and M (Cell wall/membrane/envelope biogenesis) were more abundant in cultivated ginseng soil (**Figure 5**).

### 3.4 Ginsenoside content in ginseng

**Figure 6:**
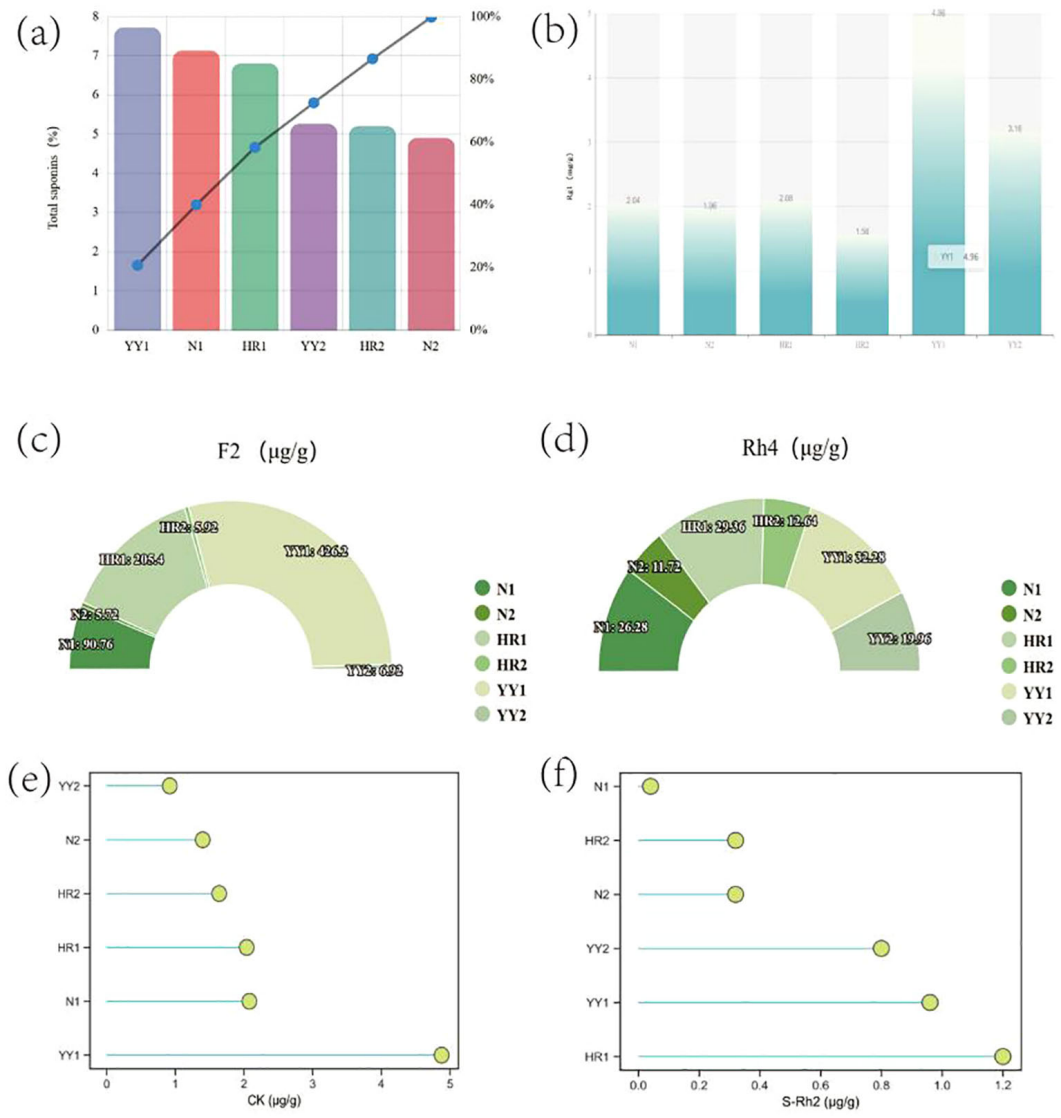
Total ginsenoside content in ginseng of different growth modes **(a)** Rg1 content in ginseng of different growth modes **(b)** F2 content in ginseng of different growth modes **(c)** Rh4 content in ginseng of different growth modes **(d)** CK content in ginseng of different growth modes **(e)** S-Rh2 content in ginseng of different growth modes **(f)**.

Among the aboveground parts of ginseng of three growth modes, wild ginseng exhibited the highest total ginsenosides content at 7.74%, while understory ginseng had the lowest at 6.82%. In the underground parts, wild ginseng again showed the highest total ginsenosides content at 5.28%, and cultivated ginseng had the lowest at 4.92% (**Figure 6a**). The Rg1 content in the aboveground and belowground parts of wild ginseng was 4.96 mg/g and 3.16 mg/g, both higher than the other two growth modes. The Rg1 content in the aboveground parts of understory ginseng and cultivated ginseng was similar, at 2.04 mg/g and 2.08 mg/g, respectively (**Figure 6b**). Among the three growth modes, the ranking of F2 content in the aboveground parts of ginseng is: wild ginseng (426.2 μg/g) > understory ginseng (205.4 μg/g) > cultivated ginseng (90.76 μg/g). For the underground parts, the F2 content ranking was: wild ginseng (6.92 μg/g) > understory ginseng (5.92 μg/g) > cultivated ginseng (5.72 μg/g) (**Figure 6c**). The Rh4 content in the aboveground and underground parts of wild ginseng was 32.28 μg/g and μg/g, respectively, both higher than those of the other two growth patterns. The Rh4 content in understory ginseng and cultivated ginseng showed little difference (**Figure 6d**). Among the aboveground parts of ginseng of the three growth modes, wild ginseng exhibited the highest CK content at 4.88 μg/g and S-Rh2 content was highest in understory ginseng at 1.2 μg/g. In the underground parts, understory ginseng showed the highest CK content at 1.64 μg/g and wild ginseng had the highest S-Rh2 content at 0.8 μg/g (**Figures 6e, f**).

### 3.5 Correlation analysis

Correlation analysis based on physical and chemical properties of soil and soil enzyme activity results revealed that S-ACP showed a significant negative correlation with AK and AP (*P* < 0.05); S-CAT showed significant positive correlations with TC, and SOC (*P* < 0.05); S-SC exhibited significant positive correlations with TC and SOC (*P* < 0.05); S-UE demonstrated significant positive correlations with TC and SOC (*P* < 0.05) (**Figure 3d**).

**Figure 7:**
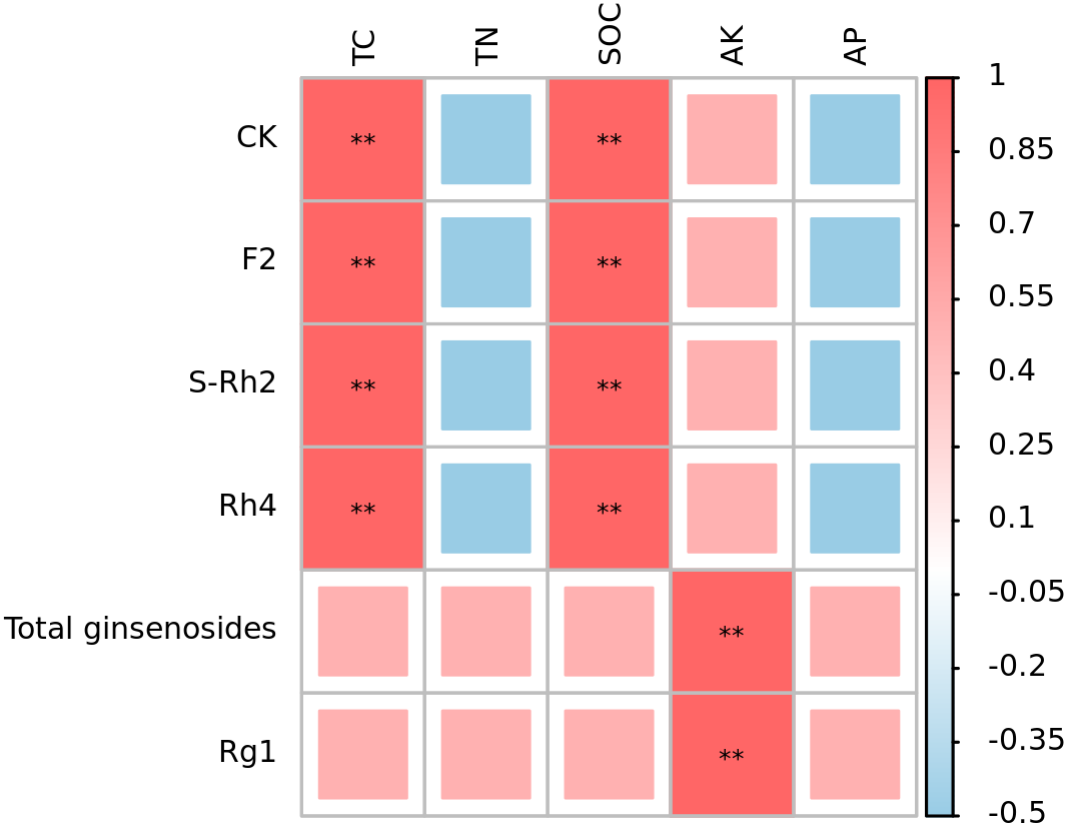
Correlation analysis between ginsenoside contents and soil physicochemical properties (a).

The correlation analysis between physical and chemical properties of soil and ginsenoside concentration revealed that total ginsenosides and Rg1 showed positive correlations with TC, SOC, TN, AK, and AP. The correlation with AK was highly significant (*P* < 0.01). CK, F2, S-Rh2, and Rh4 showed extremely significant positive correlations with TC and SOC (*P* < 0.01), positive correlations with AK, and negative correlations with TN and AP (**Figure 7**).

## 4 Discussion

### 4.1 Differences in microenvironments shaped by growth modes

Soil physicochemical properties, enzyme activities and microbial communities differed significantly among three ginseng growth modes, forming distinct rhizosphere microenvironments. Wild ginseng soil exhibited high total carbon, soil organic carbon and available potassium contents under stable and minimally disturbed forest habitats. Sufficient organic matter provided abundant substrates for microbial proliferation and community assembly. Metagenomic analysis confirmed that growth mode dominated the differentiation of soil microbial communities. Wild ginseng soil was specifically enriched in Opitutae, Verrucomicrobiae, Deltaproteobacteria and Nitrospiria, which are adapted to high-organic natural soil and possess strong nutrient transformation capacities. Microbial communities in wild ginseng soil were predominantly enriched in energy metabolism functions, consistent with its high soil enzyme activity.

Understory ginseng soil had intermediate physicochemical properties but the highest acid phosphatase activity, indicating obvious phosphorus transformation stress. The enrichment of Spartobacteria and Alphaproteobacteria, together with active microbial transcription regulation, partially alleviated phosphorus deficiency stress. However, it failed to form the stable and efficient nutrient cycling microbial community of wild ginseng soil, resulting in inferior soil biochemical properties and ginseng quality (Zhu et al., 2021).

Long-term artificial cultivation, fertilization and tillage reduced soil organic carbon storage and urease activity of cultivated ginseng soil, restricting soil nitrogen cycling. Cultivated ginseng soil was enriched in disturbance-tolerant taxa including Gemmatimonadetes, Longimicrobiia, Bacilli and Clostridia. Microbial functions were dominated by carbohydrate metabolism and environmental adaptation, lacking efficient energy accumulation and sustainable nutrient supply, which limited ginsenoside synthesis. These findings correspond to typical soil degradation caused by intensive ginseng cultivation (Zhang et al., 2008).

Correlation analysis revealed that soil catalase and sucrase activities were positively correlated with soil carbon fractions, and high-organic soil conditions optimized microbial community structure and function (Wang E. G. et al., 2024; Tuo et al., 2023). By contrast, acid phosphatase activity was negatively correlated with soil available nutrients. Phosphorus deficiency-induced enzyme variation and microbial community alteration disrupted soil nutrient cycling, leading to divergent microenvironments among different cultivation modes.

### 4.2 Soil environment and microbial communities regulate ginsenoside accumulation

Ginsenoside synthesis and accumulation are synergistically regulated by soil physicochemical properties, enzyme activities and microbial communities. Wild ginseng accumulated higher contents of total ginsenosides and key rare monomers (Rg1, F2, Rh4) than understory and cultivated ginseng. This quality advantage was attributed to not only soil physicochemical and enzymatic characteristics, but also growth-mode-dependent microbial structural and functional differentiation (Sun et al., 2023).

The high-organic substrate of wild ginseng soil continuously released available nutrients and assembled high-efficiency microbial communities. The enriched microbial energy metabolism functions synergized with high catalase and sucrase activities, establishing a stable soil nutrient cycling system. This system provides sufficient carbon skeletons and energy for ginsenoside biosynthesis, particularly promoting the accumulation of rare monomers F2 and Rh4 (Wang L. et al., 2024; Zhang et al., 2024; Yang and Jiang, 2016). Soil nutrient status and microbial functional differentiation mediated differential accumulation of ginsenoside monomers. Soil nitrogen and phosphorus facilitated the accumulation of total ginsenosides and Rg1 but inhibited the synthesis of CK, F2, S-Rh2 and Rh4. In cultivated ginseng soil, artificial disturbance caused organic matter depletion and nutrient imbalance. Disturbance-adapted microbial communities showed weak energy supply capacity. Combined with phosphorus stress, ginseng plants prioritized primary growth metabolism and reduced secondary metabolite investment, thereby decreasing rare ginsenoside accumulation (Wang et al., 2025). Understory ginseng exhibited transitional microbial and metabolic characteristics, leading to moderate ginsenoside accumulation. In conclusion, different ginseng growth modes reshaped soil physicochemical and enzymatic conditions, further driving microbial community assembly and functional differentiation. The natural wild ginseng habitat formed a high-efficiency microecological system characterized by high organic matter, high enzyme activity and active microbial energy metabolism, which determined its superior ginsenoside quality. In contrast, soil degradation and microbial functional imbalance under intensive cultivation caused the quality decline of cultivated ginseng.

## 5 Conclusion

In conclusion, different ginseng growth modes create distinct soil physicochemical, enzymatic and microbial conditions. The natural forest habitat of wild ginseng sustains high soil organic matter, strong enzyme activity and efficient microbial energy metabolism, collectively contributing to its higher ginsenoside accumulation. In comparison, long-term intensive cultivation leads to soil degradation and altered microbial functional profiles in cultivated ginseng soil, which are closely associated with reduced ginsenoside content and inferior ginseng quality. Understory ginseng exhibits intermediate soil ecological characteristics and ginsenoside accumulation levels between wild and cultivated ginseng.

This study still has certain limitations. The soil and ginseng plant samples used in this study were collected from different survey areas, and the plots exhibited significant differences in key environmental factors such as geographical origin and altitude. Although this study standardized sample testing and data analysis protocols, it was unable to completely eliminate the interference of the aforementioned confounding factors on the experimental results. The heterogeneous conditions across different plots may have influenced the interpretation of results regarding ginsenoside content variations and soil property response characteristics. Therefore, the results of this study can only reflect the overall characteristics of ginseng soil ecology and ginsenoside accumulation under different field conditions; they cannot precisely quantify the independent effects of individual environmental factors. Future studies could mitigate experimental biases caused by sample heterogeneity by controlling for individual variables, establishing homogeneous cultivation control trials, and refining the monitoring of plot environmental factors, thereby further elucidating the regulatory mechanisms of the soil environment on ginsenoside accumulation.

## Data Availability

The raw data supporting the conclusions of this article will be made available by the authors, without undue reservation.
